# From AI adaptation to innovation: how leader AI crafting shapes employee innovative behavior

**DOI:** 10.3389/fpsyg.2026.1847344

**Published:** 2026-07-09

**Authors:** Tianxing Pu, Ren Lei, Abdul Waheed Siyal, Yuanheng Li

**Affiliations:** 1 School of Digital Economics and Management, Wuxi University, Wuxi, China; 2 School of Business and Law, University of Wolverhampton, Wolverhampton, United Kingdom

**Keywords:** AI self-efficacy, AI task dependence, innovative behavior, leader AI crafting, social cognitive theory

## Abstract

As artificial intelligence reshapes work, organizations face the challenge of helping employees move beyond routine technological adaptation toward innovation. Although prior research has shown that leader AI crafting facilitates employees’ adaptation to AI, whether and how it promotes employees’ innovative behavior remains unclear. Drawing on social cognitive theory, this study examines how leader AI crafting relates to employees’ innovative behavior through AI self-efficacy and when this relationship becomes stronger. We conducted two studies. Study 1 used a two-wave field survey to examine the moderated mediation model linking leader AI crafting, AI self-efficacy, AI task dependence, and innovative behavior. Across the two studies, the results indicate that leader AI crafting enhances employees’ AI self-efficacy, which in turn is associated with higher innovative behavior. The positive effect of leader AI crafting on AI self-efficacy is stronger when AI task dependence is high, and moderated mediation effect is also supported. Study 2 used a vignette-based experiment to test the first-stage moderating role of AI task dependence in the relationship between leader AI crafting and AI self-efficacy. These findings extend the leader AI crafting literature by clarifying how employees move from adapting to AI toward innovating in AI-enabled work contexts, and by identifying AI self-efficacy and AI task dependence as key psychological and contextual mechanisms. Given the technology-industry sample in Study 1 the broader generalizability of these findings remains to be examined in future research.

## 1 Introduction

With the widespread integration of artificial intelligence (AI) into organizational settings, the boundaries, task structures, and competency requirements of work are undergoing profound transformation (Qiu et al., 2022; Tong et al., 2021). In particular, AI complicates the conventional dyadic relationship between employees and their work by introducing a more intricate triadic arrangement among humans, AI, and work tasks. As a result, both employees and organizations are increasingly compelled to renegotiate how work is performed, coordinated, and experienced. Therefore, recent studies highlights how employees actively modify their interactions with AI (Ayinde and Kirkwood, 2020; Parker and Grote, 2022) rather than view it as passive recipients of AI implementation. This line of inquiry has given rise to the concept of AI crafting, defined as employees’ proactive efforts to shape, adjust, and redefine their work practices in response to AI (Li et al., 2024).

Within this highly uncertain landscape, leaders act as critical linchpins for employee sensemaking (Liang et al., 2025). The existing studies have shown that leader AI crafting can foster employee AI crafting, AI engagement, AI helping, and employee–AI collaboration (Li et al., 2024; Liang et al., 2026). These studies have mainly explained whether leader AI crafting can help employees better adapt to AI, less is known about whether and how it extends beyond adaptation to shape more distal, higher-order, and strategically consequential employee outcomes, such as innovation. AI adaptation primarily involves learning to accommodate AI, adjusting existing work routines, and reducing resistance to technological change (Li et al., 2024; Parker and Grote, 2022). By contrast, innovative behavior in AI-enabled work contexts requires employees to generate and implement novel and useful ideas while working with AI (Janssen, 2000; Scott and Bruce, 1994). This process imposes distinct cognitive and motivational demands: employees must tolerate ambiguous AI outputs, evaluate the usefulness of algorithmic suggestions, recombine AI-generated information with domain knowledge, and persist through repeated trial and error (Bandura, 1997; Raisch and Krakowski, 2021). Therefore, mechanisms that explain AI adaptation, such as imitation, AI trust, or reduced emotional exhaustion, may not fully explain why employees move beyond routine adjustment and engage in innovation with AI (Li et al., 2024). Accordingly, we examine the relationship between leader AI crafting and employee’s innovative behaviors to assess whether leader AI crafting could stimulate the innovative behaviors in AI-enabled work contexts.

Moreover, the mechanisms through which leader AI crafting translates into employees’ innovative behavior remain insufficiently understood. Prior research has productively used social learning theory to explain how employees acquire behavioral norms by observing their leaders, emphasizing imitation, AI trust, and the alleviation of emotional exhaustion as the key pathways through which employees adapt to AI (Li et al., 2024). From this perspective, leader AI crafting may spread through vicarious learning, as employees observe, imitate, and adopt leaders’ AI-related work practices. However, moving from AI adaptation to innovative behavior requires more than the behavioral transmission of observable practices. Innovative behavior involves uncertainty, experimentation, persistence through setbacks, and the willingness to promote and implement novel ideas. Thus, employees must not only observe how leaders use AI, but also internalize the belief that they themselves are capable of using AI to generate and implement new solutions. Social cognitive theory extends the social learning logic by emphasizing this cognitive-motivational internalization process (Bandura, 1986; Compeau et al., 1999). Drawing on this perspective, we introduce AI self-efficacy as a psychological mechanism that bridges the gap between observing leader AI crafting and engaging in AI-enabled innovative behavior. AI self-efficacy refers to employees’ beliefs in their capability to effectively use AI to accomplish work tasks and cope with AI-related challenges (Lee and Cao, 2026; Zhou et al., 2025). Drawing on social cognitive theory, we argue that leader AI crafting can enhance employees’ AI self-efficacy by providing encouragement, behavioral modeling, and work-related demonstrations of how AI can be effectively integrated into task execution (Bandura, 1986, 1997). When employees develop stronger AI self-efficacy, they are more likely to persist in the face of difficulties, set more challenging goals, and adopt a more proactive problem-solving orientation rather than making passive or defensive attributions (Schunk and DiBenedetto, 2020). As a result, they become more willing and able to experiment with AI, reconfigure their work processes, and translate AI use into innovative behavior (Newman et al., 2019).

Furthermore, research on the boundary conditions of leader AI crafting remains limited, particularly with regard to the task context in which such leadership behavior is enacted. Existing studies have primarily focused on how employees interpret leaders and how they individually process AI-related information, such as through impression management or mindfulness (Li et al., 2024; Liang et al., 2026), while paying far less attention to the work context itself. Drawing on social cognitive theory, behavior results from the reciprocal interplay between environmental influences and personal factors (Bandura, 1986, 1997). In particular, we know little about whether and how the effects of leader AI crafting vary across different task environments. One important way in which task environments differ is in the extent to which task accomplishment requires close human-AI collaboration. In such environments, employees are more likely to depend on AI in carrying out their work. Therefore, neglecting employees’ dependence on AI may constrain our understanding of when and for whom leader AI crafting is most effective. Employees with higher AI task dependence are more sensitive to technological changes and more attentive to leaders’ AI-related demonstrations and encouragement (Faraj et al., 2018; Parker and Grote, 2022). Consequently, they are more likely to internalize these cues and develop stronger AI self-efficacy. By contrast, for employees with lower AI task dependence, leader AI crafting may be less salient and therefore less influential. As a result, this study identifies employees’ AI dependence as a key boundary condition.

**Figure 1:**
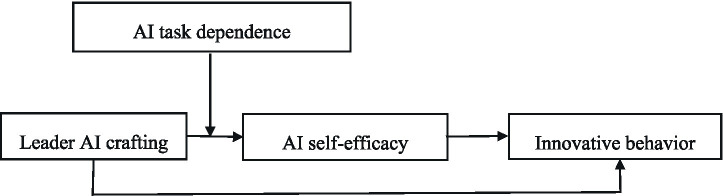
Theoretical model of leader AI crafting and Innovative behavior.

This research makes several contributions. First, it extends research on leader AI crafting by linking it to employee innovative behavior, thereby broadening its consequences beyond AI adaptation. Second, drawing on social cognitive theory, it identifies AI self-efficacy as a key mediating mechanism, shedding light on how leader AI crafting translates into employee innovation. Third, it introduces AI task dependence as an important boundary condition, offering a more nuanced understanding of when leader AI crafting is most influential. The theoretical framework articulating these relationships is presented in Figure 1.

## 2 Hypothesis development

### 2.1 Leader AI crafting and innovative behavior

Within the context of digital transformation, leader AI crafting constitutes a salient and proximal contextual cue that shapes how employees interpret, legitimize, and respond to AI in their work (Faraj et al., 2018; Parker and Grote, 2022; Raisch and Krakowski, 2021). Drawing on social cognitive theory, we argue that leader AI crafting can directly foster employees’ innovative behavior because individuals’ behavior is shaped by the reciprocal interaction between environmental cues, personal cognition, and behavioral agency (Bandura, 1986, 1997). As important agents in employees’ work environment, leaders provide social and informational cues that influence how employees evaluate opportunities, risks, and appropriate courses of action in AI-enabled work settings.

Leader AI crafting signals that AI is not merely a tool for routine adaptation but a resource for exploring new ideas, redesigning work processes, and solving problems in novel ways. When leaders proactively experiment with AI, adjust workflows around AI, and demonstrate how AI can be applied to improve work, employees are more likely to perceive AI-enabled innovation as legitimate, feasible, and valued. These cues may broaden employees’ perceived opportunity space and activate their sense of agency in using AI to improve existing work. In this way, leader AI crafting creates a social-cognitive environment in which employees are encouraged to question existing routines, recombine work resources, and develop new approaches to task accomplishment.

Moreover, leader AI crafting may stimulate employees’ proactive engagement with AI-enabled work by shaping their affective and relational responses. Leaders who actively redesign work around AI may make AI use appear more meaningful, exciting, and opportunity-oriented, thereby eliciting positive affect toward AI-enabled experimentation. Employees may also identify more strongly with leaders who display proactive, future-oriented, and competent AI use. Such positive affect and leader identification can strengthen employees’ willingness to invest effort in uncertain and exploratory work activities. Therefore, from a social cognitive perspective, leader AI crafting may directly promote innovative behavior by shaping employees’ cognitive interpretation of AI, their affective orientation toward AI-enabled work, and their agentic motivation to engage in work redesign. In line with this reasoning, prior research has shown that leader AI crafting can promote employees’ proactive AI-related behaviors, such as AI engagement (Li et al., 2024), AI helping (Tang et al., 2023), and employee–AI collaboration (Liang et al., 2026). Accordingly, we propose the following hypothesis:

*H1*: Leader AI crafting will be positively related to employees’ innovative behavior.

### 2.2 Leader AI crafting and AI self-efficacy

Drawing on social cognitive theory, we argue that leader AI crafting shapes employees’ AI self-efficacy by providing efficacy-relevant information that employees cognitively appraise when judging their own capability to use AI at work (Bandura, 1986, 1997; Schunk and DiBenedetto, 2020).

Social cognitive theory emphasizes that self-efficacy is not formed solely through exposure to external behaviors, but through individuals’ cognitive appraisal of whether a task is learnable, controllable, and attainable for themselves (Bandura, 1986, 1997; Wood and Bandura, 1989). Leader AI crafting provides such efficacy-relevant information by demonstrating how AI can be incorporated into task processes, adapted to changing work demands, and used to solve work-related problems (Faraj et al., 2018; Parker and Grote, 2022; Raisch and Krakowski, 2021). Because leaders are visible and legitimate role models in organizations, their AI-related experimentation and task redesign provide employees with credible information about the feasibility and manageability of AI-enabled work (Hughes et al., 2018; Schunk and DiBenedetto, 2020). Moreover, because these demonstrations occur within employees’ organizational and task environment, employees are more likely to regard them as applicable to their own work rather than as distant or irrelevant examples (Wood and Bandura, 1989; Parker and Grote, 2022).

Leader AI crafting may also shape AI self-efficacy through persuasive and normative cues. By actively experimenting with AI and redesigning work around AI, leaders signal that AI-enabled work practices are legitimate, valued, and worth attempting (Bandura, 1997; Hughes et al., 2018). These cues may reduce ambiguity surrounding AI use and help employees form more positive self-referent judgments about their ability to learn, apply, and manage AI effectively (Bandura, 1997; Schunk and DiBenedetto, 2020). Thus, leader AI crafting may strengthen employees’ AI self-efficacy by transforming AI from an uncertain technological demand into a more observable, interpretable, and attainable work practice. Recent empirical evidence further supports this reasoning by showing that proactive leader AI interventions can enhance employees’ positive cognitive appraisals of AI-enabled work, such as increasing AI awareness (Yang et al., 2026), fostering psychological empowerment (Ding et al., 2026), and building cognitive readiness for human–AI collaboration (Liang et al., 2026). Therefore, we propose:

*H2*: Leader AI crafting will have a positive effect on employee’s AI self-efficacy.

### 2.3 AI self-efficacy and innovative behavior

Innovative behavior entails uncertainty, experimentation, and the possibility of failure, requiring employees to move beyond established routines and persist through implementation difficulties. AI self-efficacy refers to employees’ beliefs in their capability to use AI effectively to completing work tasks and managing AI-related challenges (Bandura, 1997; Kim et al., 2024). Social cognitive theory suggests that self-efficacy shapes whether individuals initiate action, how much effort they expend, and how persistent they remain when facing obstacles (Bandura, 1986; Wood and Bandura, 1989). Accordingly, employees with high AI self-efficacy are more likely to believe that they can successfully use AI to explore new solutions, test alternative task approaches, and handle implementation difficulties arising during AI use.

This belief is particularly important for innovative behavior because innovation in AI-enabled workplaces often requires repeated experimentation, tolerance for imperfect outputs, and iterative refinement of work processes (Parker and Grote, 2022). Employees with high AI self-efficacy are more willing to engage in such experimentation because they are less likely to interpret AI-related setbacks as signs of personal inability. Instead, they are more likely to persist, adjust their prompts or usage strategies, and continue refining AI-supported solutions until novel ideas can be translated into useful implementation (Jeong and Jeong, 2025; Yang et al., 2026).

Moreover, AI self-efficacy can promote innovative behavior by encouraging proactive problem solving and task recombination. Employees who are confident in their AI-related capability are more likely to integrate AI into idea generation, information processing, and workflow redesign, thereby expanding the range of possible solutions they can develop and implement (Wang and Shao, 2024). In this sense, AI self-efficacy functions as a proximal motivational resource that enables employees not only to use AI, but also to leverage it in experimentation, adaptation, and implementation processes that underlie innovative behavior. Emerging empirical evidence in AI-enabled work contexts further supports this view, suggesting that employees with higher AI self-efficacy are more likely to engage in proactive AI use, task crafting, service innovation, and creative problem solving (Jeong and Jeong, 2025; Wang and Shao, 2024; Xu et al., 2026; Yang et al., 2026). Therefore, we propose:

*H3*: AI self-efficacy will be positively related to employees’ innovative behavior.

### 2.4 The mediating effect of AI self-efficacy

Employees’ innovative behavior is inherently uncertain, effortful, and failure-prone. As such, it is unlikely to be fully explained by external behavioral cues alone without a proximal cognitive-motivational mechanism. Drawing on social cognitive theory, we argue that AI self-efficacy represents one important mechanism through which leader AI crafting influences employees’ innovative behavior (Bandura, 1986, 1997; Schunk and DiBenedetto, 2020).

Social cognitive theory posits that environmental influences affect behavior partly by shaping individuals’ self-referent cognitions, especially efficacy beliefs about whether they can successfully perform a focal task (Bandura, 1986, 1997). In the present context, leader AI crafting provides efficacy-relevant information by visibly demonstrating that AI can be incorporated into work tasks, adapted to changing job demands, and used to improve work processes. Because leaders are salient and legitimate role models in organizations, their AI-related experimentation, task redesign, and successful application of AI provide employees with vicarious evidence that AI-enabled work is feasible and manageable (Hughes et al., 2018; Parker and Grote, 2022; Schunk and DiBenedetto, 2020). These cues reduce ambiguity surrounding AI use and strengthen employees’ beliefs that they themselves can effectively learn and apply AI in their jobs, thereby enhancing AI self-efficacy.

In turn, stronger AI self-efficacy makes employees more likely to engage in AI-enabled innovative behavior. Employees who believe they can effectively use AI are more willing to experiment with AI-supported solutions, recombine task elements, test novel work methods, and persist through technical setbacks or imperfect outputs (Jeong and Jeong, 2025). Because innovative behavior often requires employees to deviate from established routines, tolerate ambiguity, and repeatedly refine emerging ideas, AI self-efficacy serves as a proximal motivational resource that helps transform leaders’ AI-related cues into sustained experimentation and implementation (Wang and Shao, 2024; Zhu et al., 2025). Recent evidence also suggests that AI self-efficacy is closely linked to employees’ AI-related crafting and service innovation, further indicating that efficacy beliefs are a key pathway through which AI-oriented contexts and leadership cues can foster innovative outcomes (Xu et al., 2026). Therefore, we propose:

*H4*: Leader AI crafting will have a positive indirect effect on innovative behavior through AI self-efficacy.

### 2.5 The moderating role of AI task dependence

Drawing on social cognitive theory, employees’ self-beliefs and behaviors are shaped by environmental influences as well as by contextual conditions that determine the salience, relevance, and interpretability of those influences (Bandura, 1986; Schunk and DiBenedetto, 2020; Wood and Bandura, 1989). Leader AI crafting represents an important environmental cue, as it demonstrates how AI can be adjusted, integrated, and leveraged to improve work processes. AI task dependence refers to the extent to which employees must rely on AI systems to accomplish their work tasks (Berretta et al., 2023; Wang et al., 2026). We argue that employees’ AI task dependence shapes the salience and relevance of leader AI crafting.

According to social cognitive theory, efficacy beliefs can be shaped by enactive mastery experience, which is often considered a powerful source of efficacy information (Bandura, 1986, 1997). High AI task dependence might reduce the role of leader modeling because employees are more likely to accumulate direct experience with AI through their own task execution. However, this substitution logic assumes that employees’ direct AI experience provides the same type of efficacy-relevant information as leader AI crafting. In the present context, direct AI experience and leader-provided vicarious experience are more likely to be complementary than substitutive. Prior research suggests that self-efficacy develops through the cognitive integration of multiple efficacy cues, including direct mastery experience and vicarious experience, which may jointly strengthen individuals’ confidence in task accomplishment (Gist, 1987; Compeau and Higgins, 1995).

When AI task dependence is high, employees’ work is more structurally coupled with AI, making leaders’ AI-related behaviors more task-relevant and consequential for employees’ own task accomplishment. Under such conditions, employees are likely to possess more AI-related task familiarity, allowing them to better recognize the value of leaders’ AI crafting and transfer it to their own work Leader AI crafting is more likely to be interpreted not merely as a leader’s personal work adjustment, but as a transferable and actionable model for employees’ own AI-enabled work. Because employees in highly interdependent task environments repeatedly coordinate with AI during task execution, they are more likely to view leaders’ AI-related demonstrations and adaptations as directly applicable to their own work, and thus more likely to internalize these cues as efficacy-relevant information about how AI can be managed and used effectively(Parker and Grote, 2022; Wang and Long, 2025). As a result, leader AI crafting should exert a stronger positive influence on employees’ AI self-efficacy when AI task dependence is high.

By contrast, when AI task dependence is low, employees’ task accomplishment is less tightly connected to AI, and leader AI crafting may therefore be perceived as less applicable to their own work demands. In such circumstances, employees are less likely to translate leaders’ AI-related behaviors into self-referent efficacy judgments, weakening the positive effect of leader AI crafting on AI self-efficacy. Therefore, AI task dependence should strengthen the positive relationship between leader AI crafting and employees’ AI self-efficacy. Therefore, we propose:

*H5*: AI task dependence will moderate the relationship between leader AI crafting and AI self-efficacy, such that when AI task dependence is higher, the relationship between leader AI crafting and AI self-efficacy will be higher.

### 2.6 The moderated mediation role of AI task dependence

Social cognitive theory suggests that behavior is shaped through the reciprocal interplay between environmental influences and personal cognitive factors (Bandura, 1986; Wood and Bandura, 1989). Leader AI crafting represents a salient environmental cue, whereas AI self-efficacy reflects employees’ beliefs in their capability to effectively use AI in their work (Li et al., 2024). It can enhance employees’ AI self-efficacy by providing efficacy-relevant information about how AI can be integrated into work processes, adapted to task demands, and used to improve performance. In turn, employees with stronger AI self-efficacy are more likely to experiment with new approaches, persist in the face of technological challenges, and proactively leverage AI to generate and implement novel ideas, thereby engaging in innovative behavior (Bandura, 1997; Jeong and Jeong, 2025).

However, this indirect effect should not be equally strong across all work contexts. AI task dependence shapes the extent to which leader AI crafting can be translated into employees’ AI self-efficacy. When AI task dependence is high, employees’ work is more tightly coupled with AI, making leaders’ AI-related demonstrations, adaptations, and task redesign efforts more task-relevant, diagnostic, and transferable to employees’ own work. Under these conditions, employees are more likely to internalize leader AI crafting as efficacy-relevant information, thereby developing stronger AI self-efficacy (Parker and Grote, 2022; Wang and Long, 2025). As AI self-efficacy is the proximal mechanism linking leader AI crafting to innovative behavior, a stronger positive relationship between leader AI crafting and AI self-efficacy under high AI task dependence should, in turn, strengthen the indirect effect of leader AI crafting on innovative behavior.

By contrast, when AI task dependence is low, leader AI crafting is less likely to be perceived as directly applicable to employees’ own task execution, weakening its positive effect on AI self-efficacy and, consequently, its indirect effect on innovative behavior. Therefore, AI task dependence should positively moderate the indirect effect of leader AI crafting on employees’ innovative behavior via AI self-efficacy. Therefore, we propose:

*H6*: AI task dependence will moderate the indirect effect of leader AI crafting on innovative behaviors through AI self-efficacy, such that this mediated effect will be higher among employees with higher AI task dependence.

## 3 Study 1: survey study

### 3.1 Participants and procedure

To test our hypotheses, data were collected via Credamo. We restricted our sample to full-time employees who frequently used AI and related intelligent systems or software in their daily work. At the beginning of the survey, participants were informed of the study purpose, the anonymity of their responses, and the confidentiality of the data, which would be used solely for academic research. They were also told that participation was voluntary and that they could exit the survey at any time. Participants proceeded to the questionnaire only after providing informed consent.

To reduce potential common method bias, we adopted a two-wave survey design with a two-week interval. In Wave 1, we distributed questionnaires to 352 employees, measuring leader AI crafting, AI task dependence and demographic information. After removing responses that failed the attention check, were completed in an excessively short time, or exhibited obvious response patterns, 326 valid responses remained. Two weeks later, in Wave 2, these 326 respondents were invited to complete a follow-up survey measuring AI self-efficacy and employees’ innovative behavior during the previous 2 weeks. We obtained 294 valid responses in the second wave. After matching the two waves, the final sample included 294 complete matched observations.

The final matched sample of 294 represents an overall response rate of 73.5%. The demographic profile indicated a predominantly female workforce (65.6%), with the majority aged between 26 and 35 years (47.96%), followed by those aged 25 or below (25.17%). Regarding educational attainment, the sample was highly educated, with 62.9% holding a bachelor’s degree and 31.0% holding a postgraduate degree. Participants with an organizational tenure of 4 years or more constituted 54.1% of the sample. In terms of organizational rank, general employees comprised 61.6% of the sample, while managers accounted for 38.4%. Importantly, given this study’s focus on AI collaboration, the vast majority of the sample reported substantial experience interacting with AI: 75.2% had 1–3 years of AI usage experience, and 16.0% had been using AI tools for 4 years or more, thereby ensuring the sample’s appropriateness for examining AI crafting and AI self-efficacy.

### 3.2 Measurement instrument

All measurement instruments employed in this study were adapted from well-validated English language scales originally developed in Western contexts. To ensure semantic and conceptual equivalence, we implemented a translation-back-translation procedure in accordance with RW (1970) guidelines. Unless otherwise noted, all items were rated on 5-point Likert scales ranging from 1 = strongly disagree to 5 = strongly agree.

#### 3.2.1 Leader AI crafting

We measured leader AI crafting using a six-item scale developed by Li et al. (2024). A sample item is: “When working with AI robots, my supervisor introduces new approaches on his/her own to improve his/her work.” In the present study, the Cronbach’s *α* was 0.89.

#### 3.2.2 AI self-efficacy

We assessed AI self-efficacy using a three-item scale adapted from Han et al. (2025). The original scale measured generative AI (GAI) self-efficacy, and we modified the wording to align with the general AI context. A representative item is: “I feel confident in the utilization of AI even when no one is there for assistance.” During the measurement validation process, we removed ASE2 because it showed a relatively low factor loading and was less consistent with the other items in capturing employees’ confidence in using AI to accomplish work tasks. Specifically, ASE2 emphasized independent AI use without assistance, whereas our study focuses on employees’ confidence in using AI to accomplish work tasks and manage AI-related work challenges. Removing this item therefore improved the psychometric quality and conceptual coherence of the scale. After this refinement, the final three-item scale showed acceptable reliability. In the present study, Cronbach’s α was 0.71.

#### 3.2.3 AI task dependence

AI task dependence. We measured AI task dependence using a scale adapted from Van der Vegt et al. (2001). The original interpersonal task interdependence items were contextualized to capture employees’ reliance on AI systems for task accomplishment rather than mutual dependence between employees and AI systems. After assessing the measurement model, one item with a low factor loading was removed, and a four-item scale was retained for the analyses. A sample item is: “I depend on the AI system for the completion of my work.” In the present study, Cronbach’s α for the revised scale was 0.85.

#### 3.2.4 Innovative behavior

We measured innovative behavior using a six-item scale developed by Scott and Bruce (1994). Respondents were asked to indicate the extent to which they engaged in innovative behaviors, responding to items such as: “I search out new technologies, processes, techniques, and/or product ideas.” The scale showed excellent internal consistency in this study, with a Cronbach’s α of 0.86.

### 3.3 Control variables

In line with prior research, the following demographic and organizational characteristics from the time 1 survey were included as control variables: gender, age, educational attainment, organizational position, and tenure.

### 3.4 Results

#### 3.4.1 Confirmatory factor analysis and common method bias test

We first assessed the reliability and validity of the constructs. Most factor loadings exceeded 0.60, composite reliability values were above 0.70, and average variance extracted values were above or close to 0.50 (see Table 1). Although the AVE of AI self-efficacy remained below the conventional 0.50 benchmark, its composite reliability exceeded the recommended threshold. Following Fornell and Larcker (1981), convergent validity can still be considered acceptable when composite reliability is adequate. Nevertheless, although the AI self-efficacy measure can be regarded as acceptable given its adequate composite reliability, we interpret the convergent validity of AI self-efficacy with caution. Confirmatory factor analysis using Amos 23.0 showed that the four-factor model fit the data well, χ^2^ = 323.09, df = 183, χ^2^/df = 1.77, CFI = 0.95, TLI = 0.94, RMSEA = 0.05, and outperformed the alternative models (see Table 2), supporting discriminant validity.

**Table 1:** Reliability and validity testing.

Constructs	Items	Factor loading	α	CR	AVE
Leader AI crafting	LAIC1	0.79	0.89	0.89	0.58
	LAIC2	0.71			
	LAIC3	0.77			
	LAIC4	0.77			
	LAIC5	0.69			
	LAIC6	0.83			
AI self-efficacy	ASE1	0.60	0.70	0.71	0.46
	ASE2	0.55			
	ASE3	0.84			
Innovative behavior	INB1	0.73	0.86	0.87	0.52
	INB2	0.75			
	INB3	0.67			
	INB4	0.75			
	INB5	0.61			
	INB6	0.80			
AI task dependence	ATD1	0.65	0.85	0.85	0.59
	ATD2	0.70			
	ATD3	0.82			
	ATD4	0.88			

Common method bias was assessed using Harman’s single-factor test and a common method factor approach. Harman’s single-factor test showed that four factors had eigenvalues greater than 1, with the first factor explaining 36.80% of the variance, below the 50% threshold (Podsakoff et al., 2003). In addition, we used a marker variable approach to further assess common method bias. After incorporating the marker variable into the measurement model, the overall model fit improved only marginally, with CFI increasing from 0.95 to 0.97 and TLI increasing from 0.94 to 0.96. These changes were far below the 0.10 threshold, suggesting that serious common method bias was unlikely to substantially affect our findings (Meade et al., 2007).

**Table 2:** Results of confirmatory factor analysis.

Model	χ^2^	*df*	χ^2^/*df*	CFI	TLI	RMSEA
Four-factor model^a^	323.09	183	1.77	0.95	0.94	0.05
Three-factor model^b^	543.50	186	2.92	0.87	0.85	0.08
Two-factor model^c^	790.07	188	4.20	0.78	0.75	0.11
One-factor model^d^	1212.90	189	6.42	0.63	0.58	0.14
four-factor + common method factor model	200.94	127	1.58	0.97	0.96	0.05

𝐿our-factor model: leader AI crafting, AI self-efficacy, innovative behavior, and AI task dependence. ᵇThree-factor model: leader AI crafting + AI self-efficacy, innovative behavior, and AI task dependence. ᶜTwo-factor model: leader AI crafting + AI self-efficacy + innovative behavior, and AI task dependence. ᵈSingle-factor model: leader AI crafting + AI self-efficacy + innovative behavior + AI task dependence.

#### 3.4.2 Descriptive statistical analysis

Descriptive statistics and correlations are shown in Table 3. Leader AI crafting was positively and significantly associated with AI self-efficacy (*r* = 0.27, *p* < 0.01). Furthermore, AI self-efficacy was positively correlated with innovative behavior (*r* = 0.47, *p* < 0.01).

**Table 3:** Descriptive statistics and correlations for Study 1.

Variable	1	2	3	4	5	6	7	8	9	10	11
1. Gender	1.00										
2. Age	0.21**	1.00									
3. Education	0.04	0.04	1.00								
4. Position	0.17**	0.47**	0.10	1.00							
5. Tenure	0.16**	0.80**	−0.05	0.43**	1.00						
6. Industry	−0.13*	0.01	0.02	−0.06	0.01	1.00					
7. AI experience	0.07	0.22**	0.07	0.12*	0.22**	0.02	1.00				
8. Leader AI crafting	0.15**	0.12*	−0.07	0.25**	0.12*	−0.18**	0.08	1.00			
9. AI self-efficacy	0.17**	0.15*	0.00	0.11*	0.13*	−0.10*	0.10	0.25**	1.00		
10. Innovative behavior	0.18**	0.22**	0.05	0.34**	0.28**	−0.21**	0.14*	0.58**	0.45**	1.00	
11. AI task dependence	0.08	0.09	0.01	0.10	0.16*	−0.16**	0.12*	0.35**	0.13*	0.38**	1.00
M	/	32.37	4.23	1.52	2.93	3.34	2.07	4.08	4.27	3.89	3.29
SD	/	9.46	0.60	0.74	1.26	0.93	0.49	0.72	0.54	0.70	0.87

*p < 0.05, **p < 0.01.

#### 3.4.3 Hypothesis testing

We employed Model 7 of the PROCESS macro (Hayes, 2017) to examine the hypotheses. The results are shown in Table 4.

**Table 4:** The regression results of theoretical model for Study 1.

Variables	AI self-efficacy			Innovative behavior		
	*b*	SE	95% CI	*b*	SE	95% CI
Control variables						
Gender	0.22	0.12	[−0.01, 0.46]	0.03	0.09	[−0.15, 0.21]
Age	0.01	0.01	[−0.01, 0.03]	−0.02	0.01	[−0.03, 0.00]
Education level	0.07	0.10	[−0.12, 0.26]	0.15	0.07	[0.01, 0.29]
Position	−0.03	0.09	[−0.20, 0.14]	0.19	0.07	[0.06, 0.32]
Organizational tenure	0.00	0.08	[−0.14, 0.15]	0.20	0.06	[0.09, 0.31]
Industry	−0.04	0.06	[−0.16, 0.08]	−0.10	0.05	[−0.19, −0.01]
AI exposure time	0.09	0.12	[−0.14, 0.32]	0.06	0.09	[−0.11, 0.24]
Independent variable						
Leader AI crafting	0.37	0.07	[0.23, 0.52]	0.45	0.05	[0.36, 0.54]
Mediator						
AI self-efficacy				0.30	0.04	[0.21, 0.38]
Moderator						
AI task dependence	−0.01	0.06	[−0.13, 0.11]			
Interaction						
Leader AI crafting * AI task dependence	0.24	0.06	[0.13, 0.36]			
*R* ^2^	0.15			0.51		
*F*	4.85***			32.48***		

*p < 0.05, **p < 0.01, ***p < 0.001.

When testing the hypotheses, we controlled for gender, age, educational level, position, organizational tenure, industry, and AI exposure time.

Hypothesis 1 predicted that leader AI crafting would be positively related to employees’ innovative behavior. The results showed that leader AI crafting was positively associated with innovative behavior [*b* = 0.45, SE = 0.05, 95% CI (0.36, 0.54)]. Thus, Hypothesis 1 was supported.

In support of Hypothesis 2, leader AI crafting was positively associated with AI self-efficacy [*b* = 0.37, SE = 0.07, 95% CI (0.23, 0.52)]. Consistent with Hypothesis 3, AI self-efficacy was positively associated with innovative behavior [*b* = 0.30, SE = 0.04, 95% CI (0.21, 0.38)]. To test Hypothesis 4, we ran Model 4 of the PROCESS macro. The results showed that the indirect effect of leader AI crafting on innovative behavior through AI self-efficacy was significant, indirect effect = 0.06, Boot SE = 0.03, 95% Boot CI (0.02, 0.12). Therefore, Hypothesis 4 was supported.

**Figure 2:**
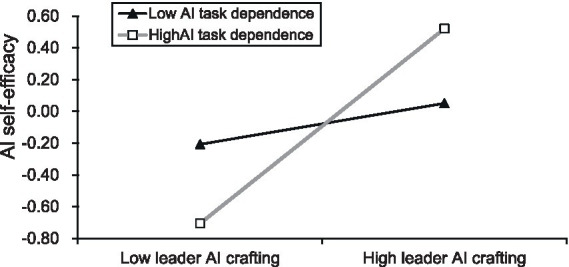
The moderating role of AI task dependence or Study 1.

To test Hypothesis 5, we created an interaction term between leader AI crafting and AI task dependence. As shown in Table 4, the interaction term had a significant positive effect on AI self-efficacy [*b* = 0.24, SE = 0.06, 95% CI (0.13, 0.36)]. Aiken et al. (1991) procedure, we plotted the interaction effect. As illustrated in Figure 2, the positive effect of leader AI crafting on AI self-efficacy was stronger when AI task dependence was high (*b* = 0.61, SE = 0.12, *p* < 0.001), and weaker when AI task dependence was low (*b* = 0.13, SE = 0.06, *p* < 0.001.). Therefore, Hypothesis 4 was supported.

Further, we tested the moderated mediation effect. The results in Table 5 show that the positive indirect effect of leader AI crafting on innovative behavior through AI self-efficacy was stronger when human-AI task dependence was high [effect = 0.18, Boot SE = 0.05, 95% CI (0.09, 0.29)], and weaker when it was low [effect = 0.04, Boot SE = 0.02, 95% CI (−0.01, 0.09)]. The difference in the indirect effects between high and low levels of human-AI task dependence was significant [diff = 0.14, Boot SE = 0.05, 95% CI (0.04, 0.24)]. The index of moderated mediation was also significant [index = 0.07, Boot SE = 0.03, 95% CI (0.02, 0.12)]. Therefore, Hypothesis 5 was supported.

**Table 5:** Moderated mediation analysis.

Variables		Effect	Boot SE	95% CI
Leader AI crafting → AI self-efficacy → innovative behavior				
AI task dependence	High	0.18	0.05	[0.09, 0.29]
	Low	0.04	0.02	[−0.01, 0.09]
	Diff	0.14	0.05	[0.04, 0.24]
Moderated mediation		0.07	0.03	[0.02, 0.12]

CI, confidence interval; LL, lower limit; UL, upper limit.

Study 1 provides field evidence for the proposed moderated mediation model linking leader AI crafting, AI self-efficacy, AI task dependence, and innovative behavior. However, we recognize that the two-wave survey design still has several limitations. In particular, AI self-efficacy and innovative behavior were both measured at Time 2, which limits our ability to draw strong conclusions about the causal ordering between the mediator and the outcome.

To remedy these issues, next we conducted a vignette-based experiment in Study 2. This experimental design allowed us to manipulate leader AI crafting and AI task dependence in a more controlled setting and to provide additional evidence for the first-stage relationship in our theoretical model.

## 4 Study 2 vignette experiment

Study 2 was designed to examine the moderating role of AI task dependence in the relationship between leader AI crafting and AI self-efficacy in a controlled experimental setting. We manipulated leader AI crafting and AI task dependence to test whether AI task dependence strengthens the positive effect of leader AI crafting on employees’ AI self-efficacy.

### 4.1 Participants and procedures

We recruited participants from business schools at two universities located in Eastern and Western China. Recruitment notices were posted to invite faculty members and students to participate in the vignette experiment. Participation was voluntary, and each participant received a small monetary reward via WeChat after completing the study.

A total of 400 participants completed the study. After excluding participants who failed the attention-check questions, the final sample consisted of 382 valid responses, yielding a valid response rate of 95.5%. Among the valid participants, 71% were female. The average age was 22.36 years (SD = 13.40).

Study 1 adopted a 2 × 2 between-subjects design, manipulating leader AI crafting (high vs. low) and AI task dependence (high vs. low). Participants were randomly assigned to one of four experimental conditions: high leader AI crafting and high AI task dependence, high leader AI crafting and low AI task dependence, low leader AI crafting and high AI task dependence, or low leader AI crafting and low AI task dependence. Before reading the scenario, participants were informed that the study was anonymous, that their responses would be kept confidential, and that the data would be used only for academic research. They were then asked to carefully read the scenario, imagine themselves as the focal employee, and answer the subsequent questions based on their genuine thoughts and feelings.

To enhance ecological validity, the scenario was designed around a digital work context that participants could understand and imagine. Specifically, participants were asked to imagine that they were a product operations specialist in a digital service company and had worked in the company for 2 years. Their main responsibilities included analyzing user feedback, organizing business data, optimizing work processes, proposing product improvement suggestions, and working with team members to implement these suggestions. The scenario further stated that the company was promoting the use of AI tools in daily work, such as using generative AI to summarize user needs, analyze data trends, generate initial proposals, optimize reports, and identify new opportunities for business improvement. The participant’s direct supervisor was described as Manager Zhang.

### 4.2 Experimental materials

After reading the common background information, participants read the manipulation materials for leader AI crafting and AI task dependence. The leader AI crafting manipulation was developed based on the conceptual meaning of leader AI crafting and related scale items.

In the high leader AI crafting condition, Manager Zhang was described as frequently and proactively exploring how to integrate AI tools into team tasks. He adjusted the use of AI according to different work requirements, such as using AI to analyze user feedback, generate proposal drafts, optimize work processes, and combine AI-generated outputs with his own professional judgment. When he found that certain work processes were inefficient, he actively tried to redesign the original workflow with AI. For example, he rearranged task procedures so that AI first completed information organization and preliminary analysis, after which team members made judgments, revisions, and improvements. Manager Zhang also actively learned new AI-related knowledge and skills and shared his experience in team meetings. He demonstrated how to ask AI more effective questions, how to evaluate the quality of AI-generated outputs, and how to transform AI results into actionable work plans.

In the low leader AI crafting condition, Manager Zhang was described as rarely taking the initiative to explore how AI tools could be integrated into team tasks. Although the company encouraged employees to use AI tools, Manager Zhang usually followed existing work procedures and seldom adjusted the use of AI according to different work requirements. When he found that certain work processes were inefficient, he typically did not try to redesign the original workflow with AI. Instead, he generally continued to use previous task procedures and rarely rearranged workflows to better leverage AI. Manager Zhang also rarely learned new AI-related knowledge and skills, seldom shared his AI-use experience in team meetings, and usually did not demonstrate how to ask AI effective questions, evaluate AI-generated outputs, or transform AI results into actionable work plans.

AI task dependence was also manipulated. The manipulation was developed based on the conceptual meaning of AI task dependence and related measurement items.

In the high AI task dependence condition, participants were told that AI tools were highly relevant to their daily tasks. They frequently needed AI to organize user feedback, analyze data trends, generate proposal drafts, summarize competitor information, and develop process improvement suggestions. The scenario further emphasized that if they could not use AI effectively, both the efficiency and quality of their work would be substantially affected. Many work outcomes needed to be developed based on the information, analysis, or initial proposals provided by AI and then revised and improved through their own professional judgment. Thus, AI had become an important tool for completing core work tasks, and their work performance largely depended on whether they could use AI effectively.

In the low AI task dependence condition, participants were told that AI tools were not closely related to their daily tasks. Their work relied more on personal experience, routine procedures, team communication, and existing business materials. Even without using AI, they could still complete most tasks smoothly. AI could occasionally provide reference information as an auxiliary tool, but it would not substantially affect their work efficiency or quality. Thus, AI was not a tool they had to rely on to complete core work tasks; instead, their work performance mainly depended on personal experience, business understanding, and team collaboration.

### 4.3 Measures

After reading the scenario, participants first completed manipulation-check items to assess whether they accurately perceived the experimental manipulations. Consistent with the Study 1, Leader AI crafting was assessed with six items adapted to the scenario, a representative item is “When working with AI robots, my supervisor introduces new approaches on his/her own to improve his/her work.” with a Cronbach’s *α* of 0.97. AI task dependence was assessed with five items adapted to the scenario. A representative item “I depend on the AI system for the completion of my work.” The Cronbach’s α for this measure is 0.91.

Consistent with the measure used in Study 1, AI self-efficacy was measured after the manipulation checks. Participants evaluated their confidence in using AI to complete work tasks and solve work-related problems in the described scenario. Sample items included “I am confident that I can effectively use AI tools to complete the work tasks described above” and “I am confident that I can use AI to solve work-related problems.” The Cronbach’s α for this scale was 0.89. This measure captured participants’ immediate efficacy beliefs regarding AI use in the experimental context. Finally, participants provided demographic information, including gender and age.

### 4.4 Experimental controls

To reduce potential order effects, the presentation order of the leader AI crafting manipulation and the AI task dependence manipulation was controlled. Specifically, within each experimental condition, some participants first read the leader AI crafting manipulation and then the AI task dependence manipulation, whereas others read the AI task dependence manipulation before the leader AI crafting manipulation.

The final valid sample sizes across the four experimental conditions were as follows: 97 participants in the high leader AI crafting and high AI task dependence condition, 94 participants in the high leader AI crafting and low AI task dependence condition, 94 participants in the low leader AI crafting and high AI task dependence condition, and 97 participants in the low leader AI crafting and low AI task dependence condition.

### 4.5 Results

#### 4.5.1 Manipulation checks

We first examined whether the manipulations of leader AI crafting and AI task dependence were effective. Independent-samples t tests showed that participants in the high leader AI crafting condition perceived significantly higher leader AI crafting than those in the low leader AI crafting condition [M_high leader AI crafting_ = 4.02, M_low leader AI crafting_ = 2.44, *t*(380) = 17.43, *p* < 0.001]. Similarly, participants in the high AI task dependence condition perceived significantly higher AI task dependence than those in the low AI task dependence condition [M_high AI task dependence_ = 4.05, M_low AI task_ dependence = 2.45, *t*(380) = 22.51, *p* < 0.001]. These results indicate that the manipulations of leader AI crafting and AI task dependence were effective.

#### 4.5.2 Hypotheses tests

Table 6 reports the descriptive statistics and correlations among the main variables in Study 2.

**Table 6:** Descriptive statistics and correlations for Study 2.

Variable	1	2	3	4	5
1. Gender	1				
2. Age	0.00	1			
3. Leader AI crafting	0.06	−0.05	1		
4. AI self-efficacy	0.04	−0.02	0.39**	1	
5. AI task dependence	−0.13*	0.02	0.02	0.03	1
*M*	0.29	22.36	0.50	3.55	0.50
SD	0.45	13.40	0.50	0.84	0.50

*p < 0.05, **p < 0.01; Gender 1 = Male, 0 = Female.

To test the moderating effect of AI task dependence, we regressed AI self-efficacy on leader AI crafting, AI task dependence, their interaction term, and the control variables. Gender and age were included as controls. To reduce potential multicollinearity, leader AI crafting and AI task dependence were standardized before creating the interaction term. The regression results are presented in Table 7.

**Table 7:** Regression results for Study 2.

Variables	AI self-efficacy		
	*b*	SE	95% CI
Control variables			
Gender	0.22	0.12	[−0.01, 0.46]
Age	0.01	0.01	[−0.01, 0.03]
Education level	0.07	0.10	[−0.12, 0.26]
Position	−0.03	0.09	[−0.20, 0.14]
Organizational tenure	0.00	0.08	[−0.14, 0.15]
Industry	−0.04	0.06	[−0.16, 0.08]
AI exposure time	0.09	0.12	[−0.14, 0.32]
Independent variable			
Leader AI crafting	0.37	0.07	[0.23, 0.52]
Mediator			
AI self-efficacy			
Moderator			
AI task dependence	−0.01	0.06	[−0.13, 0.11]
Interaction			
Leader AI crafting * AI task dependence	0.24	0.06	[0.13, 0.36]
*R* ^2^	0.15		
*F*	4.85***		

*p < 0.05, **p < 0.01, ***p < 0.001.

The results showed that leader AI crafting had a significant positive effect on AI self-efficacy [*b* = 0.39, SE = 0.05, 95% CI (0.29, 0.48)]. The interaction between leader AI crafting and AI task dependence also had a significant positive effect on AI self-efficacy [*b* = 0.10, SE = 0.05, 95% CI (0.01, 0.20)]. These results indicate that AI task dependence positively moderated the relationship between leader AI crafting and AI self-efficacy. Therefore, Hypothesis 5 was supported.

**Figure 3:**
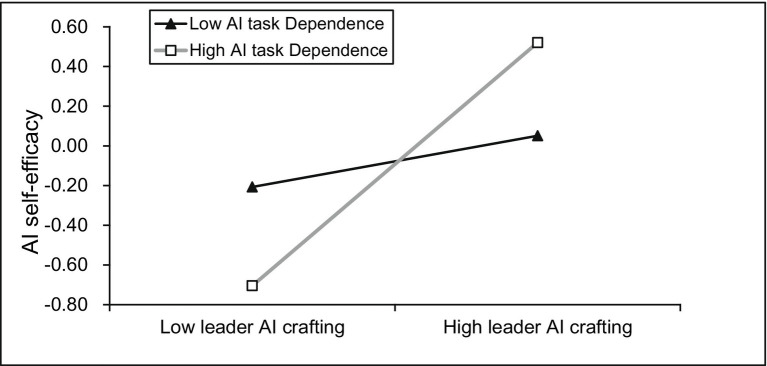
The moderating role of AI task dependence for Study 2.

To further interpret the interaction effect, we conducted simple slope analyses. The results are shown in Figure 3. The results showed that leader AI crafting was positively related to AI self-efficacy under both high and low AI task dependence. However, this relationship was stronger when AI task dependence was high, *b* = 0.50, SE = 0.07, *p* < 0.001, than when AI task dependence was low, *b* = 0.29, SE = 0.07, *p* < 0.01. This pattern supports the strengthening interpretation of AI task dependence. Thus, Study 2 supports the moderating role of AI task dependence in the relationship between leader AI crafting and AI self-efficacy.

## 5 Discussion

As organizations navigate the complexities of digital transformation, transitioning employees from routine AI adaptation to proactive innovation remains a critical theoretical and practical challenge. This study responded by investigating how leader AI crafting influences innovative behavior through AI self-efficacy and the moderating role of AI task dependence. Consistent with the proposed hypotheses, our findings revealed that leader AI crafting has a positive direct relationship with innovative behavior. AI self-efficacy mediated the relationship between leader AI crafting and innovative behavior. In addition, AI task dependence strengthened the positive relationship between leader AI crafting and AI self-efficacy, thereby amplifying the indirect relationship between leader AI crafting and innovative behavior through AI self-efficacy.

### 5.1 Theoretical implications

This study advances the literature on proactive AI adaptation by extending the consequences of leader AI crafting beyond proximal adaptation outcomes to employee innovative behavior. Prior research has primarily focused on the trickle-down effects of leader AI crafting on employees’ own AI crafting, AI engagement, AI helping, or employee–AI collaboration (Li et al., 2024; Liang et al., 2026). By examining innovative behavior as a distal outcome, our study demonstrates that leader AI crafting is not limited to facilitating technology adoption or routine AI adaptation. Rather, it may also encourage employees to generate, promote, and implement novel ideas in AI-enabled work contexts. In this way, our findings broaden the nomological network of leader AI crafting and highlight its relevance for understanding employee innovation under digital transformation. Moreover, this study enriches research on the antecedents of employee innovative behavior in digital and AI-integrated work environments. Existing studies have examined broader drivers of innovation, such as digital leadership (Zhu et al., 2025), transformational leadership (Wang and Shao, 2024), and organizational AI adoption (Wang and Long, 2025). Extending this line of inquiry, our study identifies leader AI crafting also could create conditions under which employees are more likely to pursue innovative action.

Additionally, this study clarifies the psychological mechanism through which leader AI crafting shapes employee innovative behavior by identifying AI self-efficacy as a mediator. Recent research has increasingly shown that AI-related efficacy beliefs are critical for employee creativity, innovative work behavior, and service innovation in AI-enabled workplaces (Ding et al., 2026; Jeong and Jeong, 2025; Zhang and Gao, 2026). Building on this emerging literature, we conceptualize leader AI crafting as a salient environmental cue that shapes employees’ beliefs about their capability to work effectively with AI. While prior research has emphasized more observable relational or behavioral processes such as trickle-down AI crafting and employee–AI collaboration (Li et al., 2024; Liang et al., 2026), our findings highlight the importance of employees’ internal cognitive processing in translating leader behavior into downstream outcomes. By uncovering this pathway, our study enriches understanding of how leader role modeling is translated into employees’ psychological readiness for innovation in AI-enabled workplaces. Furthermore, the remaining direct effect of leader AI crafting on innovative behavior suggests that AI self-efficacy is an important but not exhaustive mechanism. This does not diminish the role of AI self-efficacy; rather, it indicates that leader AI crafting may also shape innovative behavior through additional complementary processes. For instance, employees may learn AI-enabled innovation practices by observing leaders’ experimentation and work redesign, experience more positive affect toward AI-enabled work, or identify more strongly with leaders who demonstrate proactive AI use. These learning, affective, and relational processes may coexist with the AI self-efficacy pathway. Future research could further examine these mechanisms to provide a more complete account of how leader AI crafting fosters innovation.

Moreover, this study contributes to a more context-sensitive understanding of leader AI crafting by identifying AI task dependence as an important boundary condition. Recent work on digital work design and AI collaboration suggests that the effects of AI-related practices depend on how tightly employees’ tasks are coupled with AI and how directly AI is embedded in everyday workflows (Parker and Grote, 2022; Wang and Long, 2025; Zhu et al., 2025). Consistent with this view, our findings suggest that the influence of leader AI crafting depends on the extent to which employees’ own tasks are intertwined with AI. When AI task dependence is high, leader AI crafting becomes more salient, task-relevant, and transferable to employees’ daily work, thereby strengthening its effect on AI self-efficacy and, in turn, innovative behavior. In contrast, when such dependence is low, leader AI crafting is less likely to be internalized as efficacy-relevant information. This finding enriches the literature by showing that the effects of leader AI crafting are not uniform across work settings, but instead depend on the alignment between leader behavior and employees’ AI-related task context (Wang et al., 2026). Furthermore, this study also extends the social cognitive account of self-efficacy formation in AI-enabled work. Although direct experience and vicarious experience are both important sources of self-efficacy, they may not simply substitute for each other; rather, they can be cognitively integrated in shaping efficacy beliefs (Bandura, 1997; Gist, 1987; Gist and Mitchell, 1992). In our study, AI task dependence reflects employees’ direct experience with AI, whereas leader AI crafting provides vicarious cues about exploratory AI use and work redesign. The positive interaction between leader AI crafting and AI task dependence suggests that these two sources of efficacy information may work synergistically. Employees who depend more heavily on AI are better able to understand, interpret, and transfer leaders’ AI crafting cues to their own work. Thus, direct AI experience may strengthen, rather than replace, the effect of leader-provided vicarious experience on AI self-efficacy. This finding helps clarify how multiple sources of efficacy information jointly shape employees’ confidence in using AI in digital work contexts.

### 5.2 Practical implication

This study offers several practical implications for organizations seeking to promote innovation in AI-enabled workplaces. First, organizations should treat leader AI crafting as an important managerial capability rather than merely an individual-level technological skill. Our research demonstrates that whether a leader proactively redefines their collaboration with AI directly impacts subordinate innovation. Therefore, rather than solely focusing on general employee AI training, organizations should prioritize developing leaders’ AI crafting capabilities. Leaders should be encouraged and trained to actively and visibly demonstrate how to integrate AI into daily tasks, workflows, and decision-making processes. By doing so, they transform personal technical adaptation into a powerful role-modeling mechanism that inspires team innovation.

Secondly, organizations should focus not only on AI adoption, but also on cultivating employees’ AI self-efficacy. Our findings reveal that employees are more likely to engage in innovative behavior when they feel confident in their ability to work effectively with AI. To build such confidence, organizations can provide task-specific AI training, create opportunities for guided experimentation, offer timely feedback, and use leader demonstration and peer learning to provide employees with efficacy-enhancing experiences.

Thirdly, AI management initiatives should be highly context-specific, tailored to the degree of human-AI task dependence across different roles. Our moderated mediation results indicate that the trickle-down effect of leader AI crafting is not uniform. For positions characterized by high human-AI task dependence, leader modeling exerts a substantially stronger influence. In these domains, organizations should heavily invest in and leverage leader demonstration as a primary driver of innovation. Conversely, for roles with low AI task dependence, relying solely on leader modeling may be insufficient. In such contexts, organizations should first consider redesigning workflows to increase the structural integration of AI into daily tasks, or provide supplementary institutional support and tailored training to make AI more relevant to employees’ immediate responsibilities.

## 6 Limitations and future research directions

As with all research, this study has several limitations that open avenues for future investigation. First, although our two-study design provides complementary evidence for the proposed model, the findings should be interpreted with caution. In Study 1, AI self-efficacy and innovative behavior were measured at the same wave. Thus, although the two-wave design temporally separated leader AI crafting from the mediator and outcome, it did not fully separate the mediator from the dependent variable. This design limits our ability to make strong causal claims about the mediating role of AI self-efficacy. Future research could adopt a three-wave design or an experimental-causal-chain design to more rigorously examine the temporal ordering among leader AI crafting, AI self-efficacy, and innovative behavior.

Second, our sample was limited primarily to employees from internet technology companies in three cities in Eastern China, which constrains the generalizability of the findings. Specifically, industry and regional characteristics might influence how employees perceive leader AI crafting and engage in innovative behavior. Employees in internet technology firms may have relatively greater exposure to AI and digital technologies than employees in more traditional industries. Additional empirical research is therefore needed to verify whether our findings extend to other industries, organizational types, regions, or cultural contexts.

In addition, because innovative behavior was self-reported, social desirability bias may have influenced the results despite our efforts to ensure anonymity and confidentiality. Future research could use supervisor–subordinate matched samples in which supervisors evaluate employees’ innovative behavior, thereby reducing the potential influence of self-presentation concerns.

Finally, although this study focuses on the positive role of leader AI crafting, future research could examine its potential nonlinear effects. From a social cognitive perspective, leader AI crafting may enhance AI self-efficacy when it provides employees with attainable and task-relevant efficacy cues. However, overly complex or difficult-to-imitate leader AI crafting may set unrealistically high standards and weaken employees’ confidence in using AI. Future research could therefore examine whether leader AI crafting has an inverted U-shaped relationship with AI self-efficacy.

## Data Availability

The raw data supporting the conclusions of this article will be made available by the authors, without undue reservation.
